# Castleman's Disease Presenting as a Parotid Mass in the Pediatric Population: A Report of 2 Cases

**DOI:** 10.1155/2015/691701

**Published:** 2015-10-05

**Authors:** Sean W. Delaney, Shengmei Zhou, Dennis Maceri

**Affiliations:** ^1^Department of Otolaryngology-Head & Neck Surgery, Keck School of Medicine of USC, 1540 Alcazar Street, Suite 204, Los Angeles, CA 90033, USA; ^2^Department of Pathology, Keck School of Medicine of USC, Children's Hospital Los Angeles, Los Angeles, CA 90027, USA

## Abstract

*Introduction*. Angiofollicular lymph node hyperplasia (Castleman's disease) is a nonmalignant lymphoproliferative disorder that generally involves the lymph nodes of young adults, most commonly in the mediastinum. Rarely, Castleman's disease may present in the parotid gland. The disease can be further classified into unicentric or multicentric forms, with considerable differences in presentation, treatment, and prognosis. *Case(s)*. We present cases of two pediatric patients, aged 7 and 11, who both presented with a slow-growing, painless parotid mass. In each case, the mass was excised via a superficial parotidectomy and the diagnosis made postoperatively upon further pathologic examination. At 6 months of follow-up, both had fully intact facial nerve function and no evidence of recurrence. *Discussion*. Castleman's disease presents a diagnostic challenge in the head and neck region, as radiographic characteristics and fine needle aspiration results are often inconclusive. Definitive diagnosis requires surgical excision for pathologic examination. The unicentric form generally presents as a painless mass and can be successfully treated with complete excision. The multicentric form is associated with constitutional symptoms and its treatment remains controversial. *Conclusion*. Although rare, clinicians should be aware of both forms of Castleman's disease when creating a differential diagnosis for parotid masses.

## 1. Introduction

Dr. Benjamin Castleman first described the nonmalignant lymphoproliferative disorder of angiofollicular lymph node hyperplasia in a group of patients with thymoma-like masses of the anterior mediastinum in 1956 [1]. The entity now commonly referred to as Castleman's disease (CD) has been referred to by a number of names, including angiofollicular lymph node hyperplasia, angiomatous lymph node hamartoma, follicular lymphoreticuloma, giant lymph node hyperplasia, and benign giant lymphoma [2].

The pathogenesis of Castleman's disease remains unclear. Current hypotheses speculate chronic low grade inflammation, hamartomatous process, immunodeficient state, or pathogenic autoimmunity as potential etiology [1].

CD generally occurs in young adults [1] and has no gender predilection [3]. This disease most commonly involves mediastinal lymph nodes (60–86%) [1, 4], although it has the potential to involve any lymph node in the body. Head and neck involvement of CD is rare (6–14%) [1, 2] and often manifests as either a solitary mass under the sternocleidomastoid muscle or a mass extending superiorly from the mediastinum into the cervical region. CD involvement of the salivary gland tissues of the head and neck is exceedingly rare [2].

Clinically, CD may present in either unicentric (UCD) or multicentric (MCD) form. The UCD form generally presents as an asymptomatic painless mass and presents less frequently with compressive symptoms or cosmetic concern.

The MCD, or systemic form, has been linked to HHV-8 infection and presents with generalized lymphadenopathy, abnormal laboratory values, malaise, night sweats, hepatosplenomegaly, weight loss, and an aggressive clinical course [3]. The systemic symptoms have been attributed to IL-6 overproduction by both B-cells and the HHV-8 virus (vIL-6) [5].

CD involving the parotid gland is rare, and, to our knowledge, there are 29 reported cases in the literature of CD presenting as a parotid mass, 24 cases of UCD and 5 cases of MCD. [1–4, 6–23]. We present 2 cases of UCD that were treated over a 15-year span at Children's Hospital Los Angeles.

## 2. Case  1

**Figure 1:**
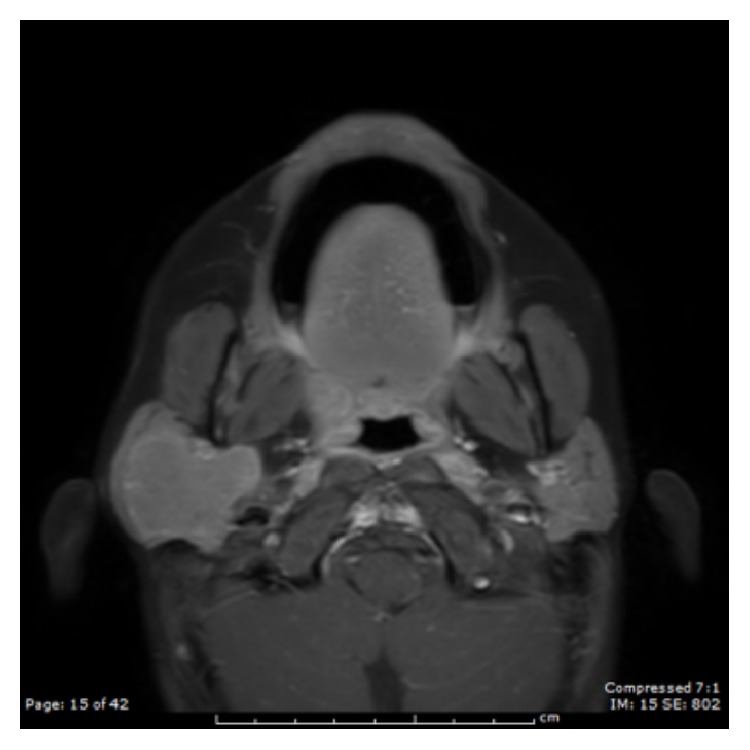
Axial T1 weight MRI (after contrast) shows a homogeneous isointense lesion.

**Figure 2:**
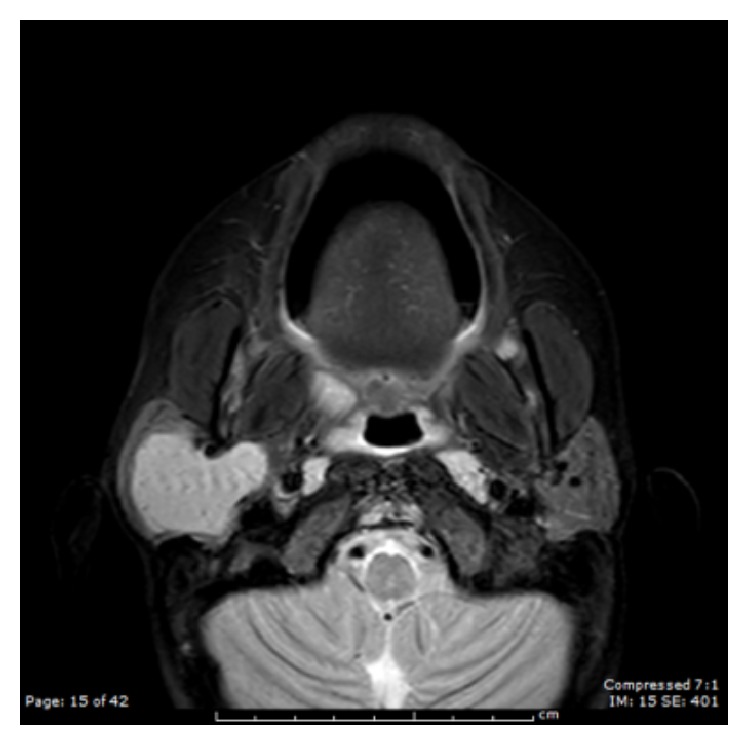
Axial T2 weight MRI shows a hyperintense lesion.

A 7-year-old male presented with a painless right facial mass for one year. On examination he had a 3 cm right parotid mass that was firm, nonpulsatile, and nontender with no overlying skin changes. His facial nerve was intact. He had no constitutional symptoms. Fine need aspiration of the mass demonstrated diffusely small lymphocytes admixed with the occasional macrophage, suggestive of a reactive lymph node. A MRI contrast study revealed a well-circumscribed homogenously T1 isointense and T2 hyperintense mass replacing nearly the entire right parotid gland and involving the deep lobe of the gland that measured 3.0 × 3.1 × 3.6 cm (Figures 1 and 2).

**Figure 3:**
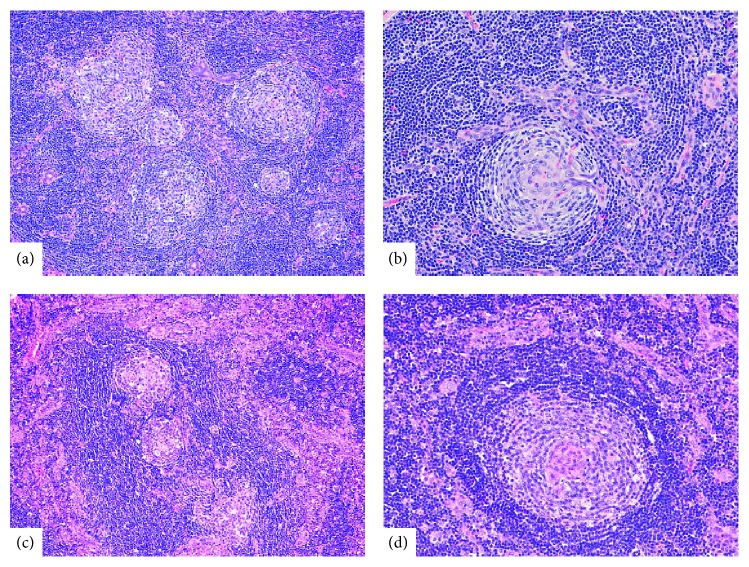
Representative microscopic images from case  1 (a and b) and case  2 (c and d). (a) Predominant lymphoid tissue surrounding variably sized salivary gland ducts. (b) Sclerotic vessels focally penetrating a geminal center, creating a “lollipop lesion.” (c) Lymphoid tissue with abnormal follicular centers. (d) A follicle with a targetoid arrangement of mantle lymphocytes and marked vascular proliferation and hyalinization in germinal center. Hematoxylin-eosin stain; original magnification: 100x for (a) and (c) and 200x for (b) and (d).

The tumor was resected via superficial parotidectomy with facial nerve dissection and had a red-tan appearance. Upon histologic examination, there was predominant lymphoid tissue surrounding variably sized salivary gland ducts. There were prominent follicles with marked vascular proliferation and hyalinization of germinal centers. Some sclerotic vessels focally penetrate germinal centers perpendicularly, creating a “lollipop lesion.” Concentric layering of peripheral small lymphocytes resembles onion-skin (Figures 3(a) and 3(b)). Background cells consist of mature lymphocytes. There was no plasma cell proliferation, granuloma, or malignancy. Surrounding nonlesional salivary tissue was unremarkable. The diagnosis of angiofollicular lymph node hyperplasia (Castleman's disease), hyaline vascular type, was made. The patient recovered well following surgery, with intact facial movement (House-Brackmann score 1/6) and no evidence of recurrence at 6 months.

## 3. Case  2

An 11-year-old female presented with a 3-year history of a slowly enlarging asymptomatic 3.5 cm right facial mass. Fine needle aspiration was nondiagnostic, showing lymphoid cells admixed with salivary gland elements. CT with contrast demonstrated a homogenously enhancing 3.5 cm × 2.5 cm right tail of parotid mass.

The tumor was also excised via superficial parotidectomy with facial nerve dissection. Intraoperatively, the tumor had a sclerotic and nodular appearance. Microscopically, sections showed lymphoid lesion of major salivary gland with variable appearance of follicles. Some follicles showed a targetoid arrangement of mantle lymphocytes and marked vascular proliferation and hyalinization in germinal centers (Figures 3(c) and 3(d)). Background cells consist of mature lymphocytes without plasma cell proliferation, granuloma, or malignant cells. The diagnosis of angiofollicular lymph node hyperplasia (Castleman's disease), hyaline vascular type, was made. Postoperatively, the facial nerve had [a] House-Brackmann score of 2/6, which recovered to House-Brackmann 1/6 at 1 week after surgery. There was no evidence of disease recurrence at 1-year follow-up.

## 4. Discussion

The differential diagnosis of a parotid mass includes salivary gland tumors, neuroma, chondroma, chondrosarcoma, fibrous tumor, reactive lymph node hyperplasia, lymphoma, branchial cleft cyst, vascular tumor or malformation, lipomatous lesion, atypical mycobacterial infection, cat-scratch disease, bacterial sialadenitis, or metastatic disease [1]. Most parotid space masses are benign.

UCD poses a diagnostic challenge, given its relative absence of symptoms and lack of specific diagnostic markers or radiographic characteristic. Radiographic findings include uniform hypoechoicity and good posterior enhancement on ultrasonography, well-defined homogenous enhancement on contrast CT, and heterogeneous isointensity on T1 and hyperintensity on T2 for MRI [3, 7]. Despite this, CD can be easily confused with other head and neck neoplasms. Imaging and fine needle aspirations are often nondiagnostic. Definitive diagnosis requires microscopic examination.

Histologically, CD has 2 distinct subtypes: (1) hyaline vascular and (2) plasma cellular [6]. Hyaline vascular is the more common subtype, comprising over 90% of tumors [2, 3, 7, 8]. This subtype is characterized by prominent lymphoid tissue with underdeveloped germinal centers surrounding salivary gland ducts of variable size, targetoid follicles within lymphoid tissue and no significant sinuses, vascular proliferation, and hyalinization of arterioles entering underdeveloped germinal centers [1]. Peripheral concentric layering of lymphocytes results in expansion of the mantle zone with an “onion-skin” appearance. There is usually no plasma cell proliferation, granuloma, or atypical mitotic figures [7]. The plasma cell subtype is marked by diffuse plasma cell proliferation between follicles and either inconspicuous or absent hyalinization [12].

Each type of CD is thought to represent the extremes of a spectrum. The plasma cell type represents an early and active stage, and the hyaline cell type represents a late and quiescent stage [3]. MCD is more commonly associated with plasma-cell histology and has a poorer prognosis due to a progressive clinical course.

The standard treatment for unicentric CD is total excision. Surgery for MCD is limited to tissue sampling for diagnosis. For patients with unresectable lesions, radiation therapy has achieved favorable responses [24, 25]. Treatment of multicentric CD remains controversial and is largely based on case reports [26]. Currently there is no consensus for the treatment of MCD and therapies include corticosteroids, chemotherapy, radiation therapy, antivirals, immunotherapy, and monoclonal antibodies [7, 27].

In summary, UCD may rarely present as a diagnostically challenging asymptomatic parotid mass. Clinicians should be aware of CD when forming a differential diagnosis, especially in the pediatric and young adult population. Surgical excision is both diagnostic and curative for UCD. MCD generally presents with systemic manifestations and treatment options for this entity remain controversial.
